# Editorial: Recent advances in our understanding of NEC pathogenesis, diagnosis, and treatment - volume II

**DOI:** 10.3389/fped.2025.1587284

**Published:** 2025-03-21

**Authors:** Xiaocai Yan, Minesh Khashu

**Affiliations:** ^1^ Division of Neonatology, Department of Pediatrics, Ann & Robert H. Lurie Children’s Hospital of Chicago, Northwestern University Feinberg School of Medicine, Chicago, IL, United States; ^2^ Division of Neonatology, Department of Paediatrics, University Hospitals Dorset, Poole, United Kingdom

**Keywords:** necrotizing enterocolitis (NEC), neonate, intestine, preterm, inflammation

**Editorial on the Research Topic**
Recent advances in our understanding of NEC pathogenesis, diagnosis and treatment - volume II

Necrotizing enterocolitis (NEC) is the leading gastrointestinal emergency in premature neonates, causing varying degrees of intestinal damage and resulting in high morbidity and mortality rates. NEC affects 2–3 per 1,000 newborns, with a mortality rate of 20%–30%, rising to 50% in surgical cases. Survivors often face long-term complications, such as short gut syndrome and neurodevelopmental delays. NEC frequently extends hospital stays for premature infants, placing significant financial and emotional strain on families as well as healthcare systems. The exact pathogenesis of NEC is not fully understood, although breast milk remains the primary feeding strategy for prevention. Due to the poorly understood underlying mechanisms, effective and specific therapeutic strategies are lacking, and the unpredictable nature of NEC continues to challenge clinical management.

This topic aims to deepen our understanding of recent advancements in the field of NEC, including tools that support clinical management, the development and validation of animal models, and the underlying pathological mechanisms driving NEC. Through this editorial, we aim to provide readers with a clearer insight into the key themes and findings discussed in this topic.

Zhang et al. developed and evaluated a risk model for adverse outcomes (intestinal perforation, the need for surgical intervention, or being too critically ill to undergo surgery) in neonates with NEC using LASSO-Cox regression analysis. The model, built on clinical and lab features, effectively identified neonates with NEC at high risk of adverse outcomes, which could help facilitate timely clinical decision-making. Going one step further, Jin and colleagues introduced a machine learning model called the CatBoost model to predict the need for surgery in infants with NEC. This model showed better predictive efficacy than traditional multifactorial logistic regression. The model incorporates birth weight, gestational age, clinical management, lab values, comorbidities, and maternal prenatal data, and its user-friendly interface could help clinicians identify infants at risk for surgery and provide guidance in terms of best place to look after such infants as well as timely surgical intervention.

Gestational age plays a critical role in NEC development, with incidence varying among early (GA: 28–<32 weeks), middle (GA: 32–<34 weeks), and late (GA: 34–<37 weeks) preterm newborns. Chen et al. found that early preterm infants had a significantly higher mortality rate and lower birth weight compared to middle and late preterms. They also observed that NEC onset and surgery timing were later in early preterms, suggesting a potential age window for NEC occurrence, corroborating previous work in this regard. Additionally, early preterms also had higher rates of comorbidities, such as sepsis and coagulopathy, leading to poorer prognosis. Identifying these factors can aid in early risk assessment and targeted interventions.

In recent years, pigs have emerged as an alternative model for NEC studies due to their closer resemblance to human intestinal development. Ragan et al. developed a new NEC model in premature piglets using only formula feeding and created a novel scoring system. Their model validation showed that formula-fed piglets had higher mortality, more severe gut injuries, higher histologic injury scores, and elevated proinflammatory cytokines compared to controls. This model could facilitate preclinical testing of new therapies and NEC prevention strategies.

An interesting retrospective study by Wang et al. revealed a reduction in NEC incidence in premature infants with a gestational age of <37 weeks during the COVID-19 period compared to pre-pandemic times. Furthermore, this study also found a shift in the blood pathogens in NEC patients between the pre-pandemic and pandemic periods, with gram-negative bacteria being dominant in the former and gram-positive bacteria being dominant in the latter. This suggests a potential effect of pandemic-related measures on the pathogen spectrum in NEC patients with bloodstream infections, as well as on the occurrence of NEC in premature infants.

The role of anemia and red blood cell transfusion (RBCT) in the development of gut injury in preterm infants remains an important area of interest and debate. Howarth and colleagues hypothesized that anemia causes gut hypoperfusion and hypoxia, leading to gut injury, while RBCT induces reperfusion injury. However, they found no significant association between hemoglobin levels, gut perfusion, or biomarkers of gut injury. Additionally, there was no significant difference in splanchnic tissue oxygenation or gut injury biomarkers before and after RBCT. Their study concluded that anemia and RBCT are not linked to tissue oxygen saturation or gut injury biomarkers in preterm infants.

Survivors of NEC often develop neurodevelopmental delays, highlighting the need for further research on the mechanisms and management strategies in this area. Martinez et al. found that NEC induces microglial activation, increased proinflammatory cytokines, and TLR4 signaling in the brain. Importantly, enteral supplementation of butyrate, a short-chain fatty acid, suppressed these pathological changes, suggesting that adding short-chain fatty acids to the diet could mitigate NEC-induced intestinal injury and neuroinflammation in preterm infants. Further studies would be very pertinent.

Intestinal alkaline phosphatase (IAP), an enzyme produced by intestinal epithelial cells, can dephosphorylate several substrates including endotoxin lipopolysaccharide (LPS). In a pilot study, Martins et al. examined the link between IAP levels and NEC in premature infants. By analyzing intestinal resection specimens from NEC patients and controls, the researchers found significantly lower IAP activity in NEC patients, suggesting a protective role for IAP. Additionally, IAP and LPS receptor Toll-like receptor-4 (TLR4) colocalized in intestinal cells, suggesting a functional relationship that could influence the inflammatory process in NEC. While the small sample size limits the study, it calls for further research to confirm these findings and help understand better the pathophysiology of NEC.

This topic has sparked significant interest within the NEC research community and hopefully we will continue to see advancements in our understanding of NEC pathogenesis as well as development of effective prevention and treatment strategies.

